# Supplementary material for: Metabolomic Analysis Points to Bioactive Lipid Species and Acireductone Dioxygenase 1 (ADI1) as Potential Therapeutic Targets in Poor Prognosis Endometrial Cancer
Source: Cancers (Basel). 2022 Jun 8;14(12):2842. doi: 10.3390/cancers14122842 (PMC9220847; doi:10.3390/cancers14122842)
Supplement: Supplementary file 1 [file cancers-14-02842-s001.zip › 1736004-supp/File S1.pdf]

Compound	HMDB ID	Class	Subclass	mass	m/z	RT	p.value	FDR	Regulation (SC vs EEC)
Phthalic acid	HMDB0002107	Benzene and substituted derivatives	Benzoic acids and derivatives	148.0255	149.0255	9.5	0.0096685	0.09476	down
Diheptyl phthalate	HMDB0251285	Benzene and substituted derivatives	Benzoic acids and derivatives	362.2476	363.2476	11.5	0.0041352	0.059558	down
Phthalic acid Mono-2-ethylhexyl Ester	HMDB0013248	Benzene and substituted derivatives	Benzoic acids and derivatives	278.1545	279.1545	9.5	0.003451	0.058653	up
Glutathione	HMDB0000125	Carboxylic acids and derivatives	Amino acids, peptides, and analo	307.0837	308.0837	0.4	0.040854	0.19345	up
Leucyl-phenylalanine	HMDB0013243	Carboxylic acids and derivatives	Amino acids, peptides, and analo	278.1661	279.1661	9.5	0.0065027	0.078944	down
L-Isoleucine	HMDB0000172	Carboxylic acids and derivatives	Amino acids, peptides, and analo	248.1468	249.1468	0.4	0.045153	0.20275	up
N-Jasmonoylisoleucine	HMDB0029391	Carboxylic acids and derivatives	Amino acids, peptides, and analo	323.2122	324.2122	9.5	0.045153	0.20275	up
Valine	HMDB0000883	Carboxylic acids and derivatives	Amino acids, peptides, and analo	117.0784	118.0784	0.4	0.033932	0.17334	up
8-iso-15-keto-PGE2	HMDB0002341	Fatty Acyls	Eicosanoids	372.2053	373.2053	7.4	0.010132	0.09476	down
Linoleyl carnitine	HMDB0006469	Fatty Acyls	Fatty acid esters	423.3364	424.3364	9.8	0.020487	0.13665	up
Tetracosatetraenoic acid	HMDB0006246	Fatty Acyls	Fatty acids and conjugates	342.2939	343.2939	7.4	0.0009416	0.030514	down
2-oxo-4-methylthiobutanoic acid	HMDB0001553	Fatty Acyls	Fatty acids and conjugates	148.0165	149.0165	11.9	0.02082	0.13665	up
Eicosatrienoic acid	HMDB0010378	Fatty Acyls	Fatty acids and conjugates	306.2528	307.2528	11.9	0.0087278	0.090507	up
alpha-Linolenic acid	HMDB0001388	Fatty Acyls	Lineolic acids and derivatives	278.2193	279.2193	11.4	0.026493	0.15008	down
FAHFA(16:0/13-O-16:0)	HMDB0112127	Fatty Acyls	Fatty acids and conjugates	548.4234	549.4234	12.9	0.010143	0.09476	down
DG(34:4)	HMDB0007530	Glycerolipids	Diradylglycerols	588.4824	589.4824	13.2	0.00064951	0.021727	down
DG(36:5)	HMDB0055974	Glycerolipids	Diradylglycerols	614.4996	615.4996	13.2	0.017212	0.12309	down
DG(36:6)	HMDB0007734	Glycerolipids	Diradylglycerols	612.4706	613.4706	13.1	0.013272	0.11253	down
DG(37:6)	HMDB0007764	Glycerolipids	Diradylglycerols	626.4929	627.4929	13.2	0.025968	0.15008	down
DG(40:4)	HMDB0056054	Glycerolipids	Diradylglycerols	672.5836	673.5836	15.4	0.024363	0.1494	down
DG(40:8)	HMDB0056354	Glycerolipids	Diradylglycerols	664.5056	665.5056	13.2	0.015826	0.12123	down
DG(44:11)	HMDB0007759	Glycerolipids	Diradylglycerols	736.4961	737.4961	10.9	0.033685	0.17334	down
DG(i-28:0)	HMDB0093347	Glycerolipids	Diradylglycerols	534.4314	535.4314	12.9	0.0056723	0.070026	down
DG(i-33:0)	HMDB0094135	Glycerolipids	Diradylglycerols	582.5331	583.5331	14.1	0.022169	0.13943	down
MG(18:1)	HMDB0011536	Glycerolipids	Monoradylglycerols	356.293	357.293	11.3	0.013272	0.11253	down
MG(18:3)	HMDB0011570	Glycerolipids	Monoradylglycerols	374.2435	375.2435	10.4	0.0056723	0.070026	down
MG(20:5)	HMDB0011580	Glycerolipids	Monoradylglycerols	398.2496	399.2496	11.7	0.006547	0.078944	down
MG(22:0)	HMDB0011581	Glycerolipids	Monoradylglycerols	452.3387	453.3387	5.9	0.011262	0.1022	down
MG(22:6)	HMDB0011587	Glycerolipids	Monoradylglycerols	384.2849	385.2849	10.4	0.0021001	0.042052	down
MG(i-17:0)	HMDB0072852	Glycerolipids	Monoradylglycerols	366.2718	367.2718	11.4	0.026493	0.15008	down
CDP-DG(38:4)	HMDB0115989	Glycerophospholipids	CDP-glycerols	1067.5164	1068.5164	13.3	0.045361	0.20275	up
LysoPA(18:1)	HMDB0007851	Glycerophospholipids	Glycerophosphates	418.2675	419.2675	10.5	0.0000217	0.0037419	down
LysoPA(20:2)	HMDB0114758	Glycerophospholipids	Glycerophosphates	462.2957	463.2957	10.6	0.00027798	0.012533	down
LysoPA(20:3)	HMDB0114747	Glycerophospholipids	Glycerophosphates	498.2271	499.2271	6.9	0.013508	0.11253	down
LysoPA(22:1)	HMDB0114750	Glycerophospholipids	Glycerophosphates	474.32	475.32	8.2	0.0041352	0.059558	down
LysoPA(P-16:0)	HMDB0011154	Glycerophospholipids	Glycerophosphates	376.2554	377.2554	10.9	0.044511	0.20275	down
PA(28:1)	HMDB0114795	Glycerophospholipids	Glycerophosphates	572.4021	573.4021	12.6	0.025604	0.15008	down
PA(29:1)	HMDB0114813	Glycerophospholipids	Glycerophosphates	604.4157	605.4157	10.7	0.0021604	0.042052	down
PA(30:2)	HMDB0114853	Glycerophospholipids	Glycerophosphates	616.4202	617.4202	10.8	0.00015155	0.0098224	down
PA(36:6)	HMDB0114792	Glycerophospholipids	Glycerophosphates	692.4684	693.4684	10.8	0.0056723	0.070026	down
PA(36:7)	HMDB0115011	Glycerophospholipids	Glycerophosphates	690.4537	691.4537	10.5	0.0037003	0.059558	down
PA(38:5)	HMDB0115151	Glycerophospholipids	Glycerophosphates	704.4727	705.4727	10.9	0.0076209	0.084073	down
PA(40:7)	HMDB0114943	Glycerophospholipids	Glycerophosphates	746.4967	747.4967	12.5	0.021704	0.13943	up
PA(44:9)	HMDB0115344	Glycerophospholipids	Glycerophosphates	780.5217	781.5217	10.9	0.019748	0.13665	down
PA(46:7)	HMDB0115479	Glycerophospholipids	Glycerophosphates	830.5771	831.5771	12.2	0.0076209	0.084073	down
PA(48:0)	HMDB0115453	Glycerophospholipids	Glycerophosphates	854.7351	855.7351	14.9	0.0000866	0.0081641	down
PA(48:1)	HMDB0115480	Glycerophospholipids	Glycerophosphates	852.7199	853.7199	14.6	0.0014506	0.035815	down
PA(48:2)	HMDB0115481	Glycerophospholipids	Glycerophosphates	890.6926	891.6926	14.3	0.0098617	0.09476	down
PA(i-27:0)	HMDB0115801	Glycerophospholipids	Glycerophosphates	560.3887	561.3887	10.7	0.00097426	0.030615	down
PA(P-34:2)	HMDB0011155	Glycerophospholipids	Glycerophosphates	656.503	657.503	13	0.02976	0.16074	down
LysoPC(20:1)	HMDB0010391	Glycerophospholipids	Glycerophosphocholines	531.3782	532.3782	10.3	0.00064951	0.021727	down
LysoPC(22:2)	HMDB0010400	Glycerophospholipids	Glycerophosphocholines	575.4025	576.4025	10.4	0.0021001	0.042052	down
LysoPC(28:1)	HMDB0029221	Glycerophospholipids	Glycerophosphocholines	643.487	644.487	10.8	0.045361	0.20275	down
LysoPC(P-18:1)	HMDB0010408	Glycerophospholipids	Glycerophosphocholines	487.3517	488.3517	10.2	0.022018	0.13943	down
PC(34:5)	HMDB0007885	Glycerophospholipids	Glycerophosphocholines	751.5083	752.5083	10.6	0.0041352	0.059558	down
PC(38:6)	HMDB0008212	Glycerophospholipids	Glycerophosphocholines	805.5721	806.5721	13.5	0.035678	0.17618	down
LysoPE(24:1)	HMDB0011528	Glycerophospholipids	Glycerophosphoethanolamines	545.392	546.392	10.7	0.010132	0.09476	down
LysoPE(O-16:3)		Glycerophospholipids	Glycerophosphoethanolamines	433.252	434.252	10.7	0.017016	0.12309	down
PE(36:1)	HMDB0009057	Glycerophospholipids	Glycerophosphoethanolamines	745.5744	746.5744	13.5	0.0044838	0.062834	down
PE(38:1)	HMDB0008999	Glycerophospholipids	Glycerophosphoethanolamines	773.6007	774.6007	13.7	0.0035067	0.058653	down
PE(42:9)	HMDB0009694	Glycerophospholipids	Glycerophosphoethanolamines	795.534	796.534	10.6	0.01991	0.13665	down
PE(O-35:6)	HMDB0008873	Glycerophospholipids	Glycerophosphoethanolamines	707.481	708.481	10.5	0.0021001	0.042052	down
LysoPG(18:1)	HMDB0240602	Glycerophospholipids	Glycerophosphoglycerols	492.3069	493.3069	10.2	0.0000217	0.0037419	down
PG(38:7)	HMDB0116631	Glycerophospholipids	Glycerophosphoglycerols	792.5243	793.5243	11	0.022018	0.13943	down
PG(40:5)	HMDB0010612	Glycerophospholipids	Glycerophosphoglycerols	824.5453	825.5453	10.9	0.0029877	0.052513	down
PG(40:8)	HMDB0010659	Glycerophospholipids	Glycerophosphoglycerols	800.4891	801.4891	10.6	0.0000217	0.0037419	down
PG(42:9)	HMDB0116744	Glycerophospholipids	Glycerophosphoglycerols	844.5149	845.5149	10.7	0.0012528	0.03453	down
PG(i-28:0)	HMDB0116664	Glycerophospholipids	Glycerophosphoglycerols	648.4432	649.4432	10.8	0.0005173	0.021458	down
PG(i-30:2)	HMDB0116654	Glycerophospholipids	Glycerophosphoglycerols	712.4383	713.4383	10.5	0.0000217	0.0037419	down
PGP(40:4)	HMDB0013513	Glycerophospholipids	Glycerophosphoglycerols	888.5384	889.5384	10.7	0.0028316	0.052435	down
PGP(i-30:0)	HMDB0116542	Glycerophospholipids	Glycerophosphoglycerols	756.4626	757.4626	10.6	0.0000433	0.0064146	down
PGP(i-37:0)	HMDB0116565	Glycerophospholipids	Glycerophosphoglycerols	872.5459	873.5459	11.2	0.033685	0.17334	down
PIP2(36:1)	HMDB0010063	Glycerophospholipids	Glycerophosphoinositol phosphat	1006.5233	1007.5233	10.6	0.02082	0.13665	up
LysoPI(18:2)	HMDB0240597	Glycerophospholipids	Glycerophosphoinositols	578.2943	579.2943	9.5	0.003452	0.058653	down
PS(38:3)	HMDB0112598	Glycerophospholipids	Glycerophosphoserines	813.5753	814.5753	9.5	0.0028304	0.052435	down
PS(40:1)	HMDB0112400	Glycerophospholipids	Glycerophosphoserines	883.5858	884.5858	10.7	0.049943	0.22133	down
Kynurenine	HMDB0000684	Organooxygen compounds	Carbonyl compounds	208.0856	209.0856	0.8	0.027929	0.15324	up
3-hydroxypristanic acid	HMDB0061651	Prenol lipids	Diterpenoids	296.2762	297.2762	12.1	0.00015155	0.0098224	down
Pristanic acid	HMDB0000795	Prenol lipids	Diterpenoids	298.2912	299.2912	12.4	0.0041352	0.059558	down
2'-Deoxyguanosine 5'-monophosphate / adenosine monophosphate	HMDB0001044	Purine nucleotides	Purine deoxyribonucleotides/Puri	347.0631	348.0631	0.6	0.0021898	0.042052	up
SM(d40:1)	HMDB0012092	Sphingolipids	Phosphosphingolipids	786.6773	787.6773	14.4	0.0056723	0.070026	down
SM(d40:2)	HMDB0240633	Sphingolipids	Phosphosphingolipids	784.6655	785.6655	14.2	0.0076209	0.084073	down
SM(d41:1)	HMDB0011695	Sphingolipids	Phosphosphingolipids	800.6941	801.6941	14.5	0.0041352	0.059558	down
SM(d41:2)	HMDB0240614	Sphingolipids	Phosphosphingolipids	798.6723	799.6723	14.3	0.0002598	0.012246	down
SM(d42:0)	HMDB0012094	Sphingolipids	Phosphosphingolipids	838.7151	839.7151	14.5	0.0002598	0.012246	down
SM(d42:1)	HMDB0011697	Sphingolipids	Phosphosphingolipids	796.6668	797.6668	14.1	0.034987	0.17359	down
SM(d42:2)	HMDB0240635	Sphingolipids	Phosphosphingolipids	812.6951	813.6951	14.4	0.0056723	0.070026	down
SM(d43:1)	HMDB0240673	Sphingolipids	Phosphosphingolipids	828.7289	829.7289	14.8	0.017212	0.12309	down
SM(d43:2)	HMDB0011699	Sphingolipids	Phosphosphingolipids	826.7039	827.7039	14.6	0.0000217	0.0037419	down
SM(d44:2)	HMDB0013461	Sphingolipids	Phosphosphingolipids	840.7301	841.7301	14.7	0.0002598	0.012246	down
Chenodeoxycholic acid 3-sulfate	HMDB0002586	Steroids and steroid derivatives	Bile acids, alcohols and derivative	472.2704	473.2704	11.7	0.0076209	0.084073	down
12-Ketodeoxycholic acid	HMDB0000328	Steroids and steroid derivatives	Bile acids, alcohols and derivative	390.2796	391.2796	11.9	0.026276	0.15008	up
7-Ketodeoxycholic acid	HMDB0000391	Steroids and steroid derivatives	Bile acids, alcohols and derivative	4289.2434	429.2434	11.9	0.02082	0.13665	up
1021.5268_11.5				1020.5268	1021.5268	11.5	0.037003	0.18015	down
1061.6003_8.7				1060.6003	1061.6003	8.7	0.02976	0.16074	down

1074.4623_12.9	1073.4623	1074.4623	12.9	0.020487	0.13665	up
109.0937_3.7	108.0937	109.0937	3.7	0.015965	0.12123	down
111.1038_3.3	110.1038	111.1038	3.3	0.0056723	0.070026	down
1119.6414_9.1	1118.6414	1119.6414	9.1	0.0000866	0.0081641	down
149.0175_9.5	148.0175	149.0175	9.5	0.0087278	0.090507	up
161.0715_3.1	160.0715	161.0715	3.1	0.020487	0.13665	up
167.0823_0.8	166.0823	167.0823	0.8	0.043474	0.20036	down
198.9881_1.5	197.9881	198.9881	1.5	0.020467	0.13665	down
201.1752_10	200.1752	201.1752	10	0.03336	0.17334	down
205.0781_9.5	204.0781	205.0781	9.5	0.0087278	0.090507	up
205.0898_9.5	204.0898	205.0898	9.5	0.015615	0.12123	down
219.1665_9.9	218.1665	219.1665	9.9	0.0098617	0.09476	down
230.2406_8.1	229.2406	230.2406	8.1	0.0042499	0.060372	down
246.1644_1.8	245.1644	246.1644	1.8	0.03336	0.17334	down
254.1293_5.9	253.1293	254.1293	5.9	0.027929	0.15324	down
258.2726_9.1	257.2726	258.2726	9.1	0.011333	0.1022	down
265.2412_11.3	264.2412	265.2412	11.3	0.0012343	0.03453	down
271.261_12	270.261	271.261	12	0.02976	0.16074	down
304.2932_8.6	303.2932	304.2932	8.6	0.024492	0.1494	down
312.3223_12.2	311.3223	312.3223	12.2	0.0003804	0.016437	down
318.2941_9	317.2941	318.2941	9	0.025968	0.15008	down
327.1758_8.1	326.1758	327.1758	8.1	0.034448	0.17341	down
341.1097_2.7	340.1097	341.1097	2.7	0.0041128	0.059558	down
341.2549_10.3	340.2549	341.2549	10.3	0.00064951	0.021727	down
350.2538_11.1	349.2538	350.2538	11.1	0.007424	0.084073	up
350.754_11.1	349.754	350.754	11.1	0.034446	0.17341	up
367.1421_0.7	366.1421	367.1421	0.7	0.0087278	0.090507	up
367.2076_7.4	366.2076	367.2076	7.4	0.012724	0.11182	down
369.232_6.9	368.232	369.232	6.9	0.012607	0.11173	down
373.2266_5	372.2266	373.2266	5	0.013272	0.11253	down
378.7853_11.7	377.7853	378.7853	11.7	0.0021816	0.042052	up
387.2404_6	386.2404	387.2404	6	0.0056723	0.070026	down
394.2944_11.9	393.2944	394.2944	11.9	0.020487	0.13665	up
394.7193_7.4	393.7193	394.7193	7.4	0.010132	0.09476	down
395.2189_7.4	394.2189	395.2189	7.4	0.017016	0.12309	down
397.2698_11.7	396.2698	397.2698	11.7	0.025961	0.15008	up
401.09_9.5	400.09	401.09	9.5	0.022185	0.13943	down
401.2632_6.9	400.2632	401.2632	6.9	0.0076209	0.084073	down
409.7571_10.6	408.7571	409.7571	10.6	0.0021816	0.042052	down
412.7766_13.6	411.7766	412.7766	13.6	0.0087278	0.090507	up
414.3134_10.5	413.3134	414.3134	10.5	0.042169	0.19877	down
415.2388_11.4	414.2388	415.2388	11.4	0.013885	0.11253	down
416.7644_11	415.7644	416.7644	11	0.0002598	0.012246	down
424.353_10.5	423.353	424.353	10.5	0.034987	0.17359	down
425.1787_10.5	424.1787	425.1787	10.5	0.012607	0.11173	down
431.7713_10.7	430.7713	431.7713	10.7	0.040224	0.19134	down
433.3314_12.4	432.3314	433.3314	12.4	0.010132	0.09476	down
437.7031_7.4	436.7031	437.7031	7.4	0.037667	0.18084	down
438.7803_11	437.7803	438.7803	11	0.017212	0.12309	down
441.1492_6	440.1492	441.1492	6	0.022018	0.13943	down
449.2818_10.2	448.2818	449.2818	10.2	0.010132	0.09476	down
453.3369_8.2	452.3369	453.3369	8.2	0.0029539	0.052513	down
453.3389_2.7	452.3389	453.3389	2.7	0.046193	0.20559	down
453.8432_8.2	452.8432	453.8432	8.2	0.0021001	0.042052	down
464.835_8.2	463.835	464.835	8.2	0.03795	0.18136	down
465.8122_11	464.8122	465.8122	11	0.043474	0.20036	down
466.5275_11.7	465.5275	466.5275	11.7	0.031064	0.1652	down
468.7948_11	467.7948	468.7948	11	0.037003	0.18015	down
471.3477_4.2	470.3477	471.3477	4.2	0.0041352	0.059558	down
472.3156_8.2	471.3156	472.3156	8.2	0.0098617	0.09476	down
473.338_10.6	472.338	473.338	10.6	0.0000866	0.0081641	down
475.7955_10.8	474.7955	475.7955	10.8	0.013508	0.11253	down
485.3624_4.9	484.3624	485.3624	4.9	0.0011978	0.03453	down
495.2666_11.9	494.2666	495.2666	11.9	0.020487	0.13665	up
512.4092_10.6	511.4092	512.4092	10.6	0.017212	0.12309	down
513.2733_8.3	512.2733	513.2733	8.3	0.020487	0.13665	up
515.2018_6.9	514.2018	515.2018	6.9	0.013885	0.11253	down
517.3645_10.6	516.3645	517.3645	10.6	0.00015155	0.0098224	down
520.3271_5.3	519.3271	520.3271	5.3	0.037253	0.18052	down
522.5968_12.1	521.5968	522.5968	12.1	0.045361	0.20275	up
529.8302_11.1	528.8302	529.8302	11.1	0.025144	0.15008	down
534.333_11.1	533.333	534.333	11.1	0.037667	0.18084	down
537.3314_10.3	536.3314	537.3314	10.3	0.0002598	0.012246	down
549.4311_13	548.4311	549.4311	13	0.016015	0.12123	down
549.9556_11.7	548.9556	549.9556	11.7	0.033791	0.17334	up
550.6221_12.3	549.6221	550.6221	12.3	0.0000866	0.0081641	down
556.4331_10.7	555.4331	556.4331	10.7	0.034987	0.17359	down
557.1855_6	556.1855	557.1855	6	0.02663	0.15008	up
557.5207_14	556.5207	557.5207	14	0.031064	0.1652	down
567.2764_5.9	566.2764	567.2764	5.9	0.033685	0.17334	down
570.8598_11.2	569.8598	570.8598	11.2	0.030195	0.16224	down
575.2704_5.9	574.2704	575.2704	5.9	0.022018	0.13943	down
581.3576_10.4	580.3576	581.3576	10.4	0.036505	0.17941	down
585.5519_14.3	584.5519	585.5519	14.3	0.026493	0.15008	down
595.8757_11.2	594.8757	595.8757	11.2	0.0087278	0.090507	up
597.8952_11.2	596.8952	597.8952	11.2	0.011012	0.10196	down
599.2244_10.6	598.2244	599.2244	10.6	0.025968	0.15008	down
600.4616_10.7	599.4616	600.4616	10.7	0.043474	0.20036	down
605.2764_5.9	604.2764	605.2764	5.9	0.043474	0.20036	down
610.5388_13.2	609.5388	610.5388	13.2	0.024492	0.1494	down
614.8841_11.2	613.8841	614.8841	11.2	0.011333	0.1022	down
620.4301_10.4	619.4301	620.4301	10.4	0.00015155	0.0098224	down
623.2668_5.9	622.2668	623.2668	5.9	0.0011355	0.03453	down
627.4276_7.9	626.4276	627.4276	7.9	0.022569	0.14099	down
631.4526_12.5	630.4526	631.4526	12.5	0.02663	0.15008	up
639.4815_13.2	638.4815	639.4815	13.2	0.01389	0.11253	down
641.4591_9.4	640.4591	641.4591	9.4	0.03336	0.17334	down

653.3796_10.8	652.3796	653.3796	10.8	0.0098617	0.09476	down
655.4849_12.9	654.4849	655.4849	12.9	0.00019448	0.011863	down
659.2847_11.5	658.2847	659.2847	11.5	0.0041001	0.059558	down
668.6204_12.2	667.6204	668.6204	12.2	0.00015155	0.0098224	down
685.512_7.4	684.512	685.512	7.4	0.0021001	0.042052	down
69.0584_3.3	68.0584	69.0584	3.3	0.027929	0.15324	down
696.4294_6.4	695.4294	696.4294	6.4	0.024363	0.1494	down
696.6486_12.4	695.6486	696.6486	12.4	0.034448	0.17341	down
706.4664_7.4	705.4664	706.4664	7.4	0.00064951	0.021727	down
706.9627_7.4	705.9627	706.9627	7.4	0.0021604	0.042052	down
710.9616_7.4	709.9616	710.9616	7.4	0.01617	0.12151	down
711.4633_7.4	710.4633	711.4633	7.4	0.007424	0.084073	down
717.4817_6.8	716.4817	717.4817	6.8	0.045077	0.20275	down
735.4814_10.6	734.4814	735.4814	10.6	0.0012653	0.03453	down
741.4174_7.4	740.4174	741.4174	7.4	0.01389	0.11253	down
777.4526_7.4	776.4526	777.4526	7.4	0.0056723	0.070026	down
779.4048_7.4	778.4048	779.4048	7.4	0.024682	0.14968	down
793.444_7.4	792.444	793.444	7.4	0.015965	0.12123	down
805.0151_13.4	804.0151	805.0151	13.4	0.020487	0.13665	up
823.6836_14.2	822.6836	823.6836	14.2	0.027929	0.15324	down
825.6865_14.4	824.6865	825.6865	14.4	0.0076209	0.084073	down
831.4429_7.4	830.4429	831.4429	7.4	0.0021001	0.042052	down
833.0487_13.5	832.0487	833.0487	13.5	0.014177	0.11397	down
847.4222_7.4	846.4222	847.4222	7.4	0.015965	0.12123	down
852.4565_7.4	851.4565	852.4565	7.4	0.02681	0.15028	down
855.4121_7.4	854.4121	855.4121	7.4	0.015965	0.12123	down
865.7169_14.5	864.7169	865.7169	14.5	0.0014506	0.035815	down
867.7357_14.8	866.7357	867.7357	14.8	0.00064951	0.021727	down
869.7587_15.1	868.7587	869.7587	15.1	0.013272	0.11253	down
873.6244_12.3	872.6244	873.6244	12.3	0.017212	0.12309	down
877.7327_14.5	876.7327	877.7327	14.5	0.0041352	0.059558	down
879.7479_14.7	878.7479	879.7479	14.7	0.0029877	0.052513	down
881.763_14.9	880.763	881.763	14.9	0.0000217	0.0037419	down
883.78_15.3	882.78	883.78	15.3	0.0014506	0.035815	down
887.4749_7.2	886.4749	887.4749	7.2	0.0056723	0.070026	down
895.7644_15.1	894.7644	895.7644	15.1	0.0014506	0.035815	down
907.7667_15	906.7667	907.7667	15	0.0056723	0.070026	down
909.7818_15.4	908.7818	909.7818	15.4	0.00064951	0.021727	down
917.57_11.2	916.57	917.57	11.2	0.015965	0.12123	down
923.6378_12.2	922.6378	923.6378	12.2	0.025961	0.15008	down
