# Supplementary material for: Metabolomic Analysis Points to Bioactive Lipid Species and Acireductone Dioxygenase 1 (ADI1) as Potential Therapeutic Targets in Poor Prognosis Endometrial Cancer
Source: Cancers (Basel). 2022 Jun 8;14(12):2842. doi: 10.3390/cancers14122842 (PMC9220847; doi:10.3390/cancers14122842)

Figure-S3 A

ADI 1

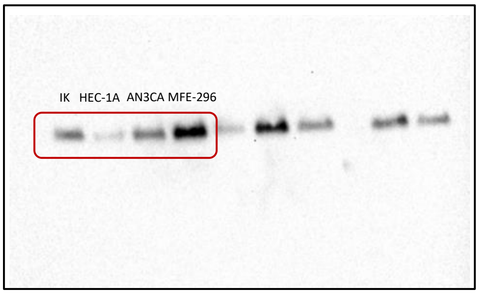

GAPDH

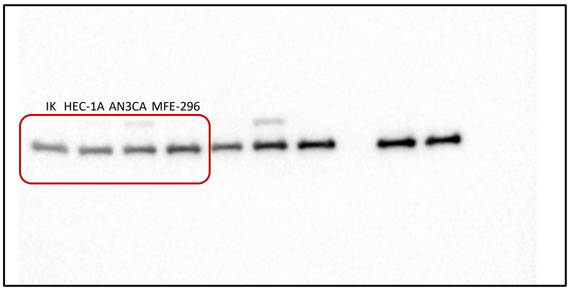

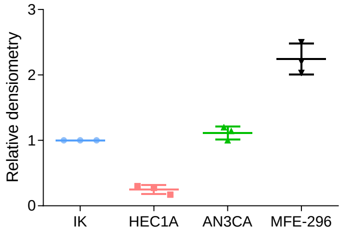

The quantification of the densiometric intensity of each western blot ADI1 band has been calculated from three western blot plots from three independent experiments. GAPDH was used as loading control.

Figure-S3 B (Left)

ADI 1

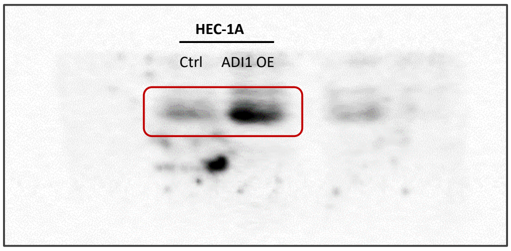

GAPDH

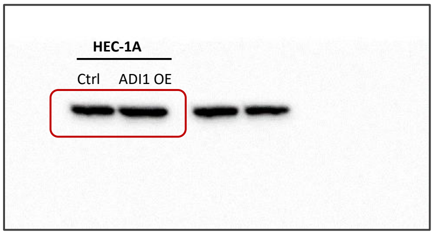

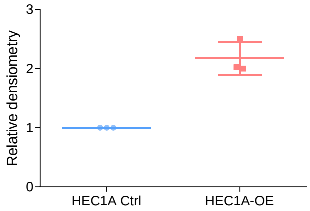

The quantification of the densiometric intensity of each western blot ADI1 band has been calculated from three western blot plots from three independent experiments. GAPDH was used as loading control.

Figure-S3 B (Right)

ADI 1

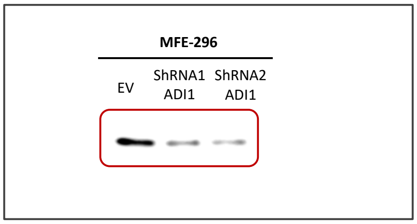

GAPDH

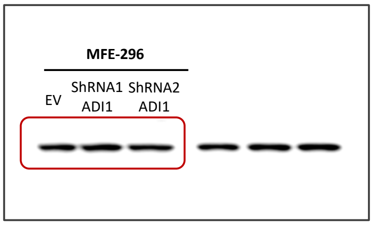

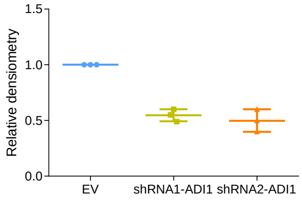

The quantification of the densiometric intensity of each western blot ADI1 band has been calculated from three western blot plots from three independent experiments. GAPDH was used as loading control.

Colorimetric- Uncropped western blots

Figure-S3 A

ADI 1

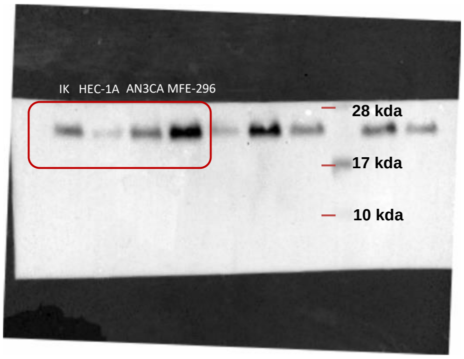

GAPDH

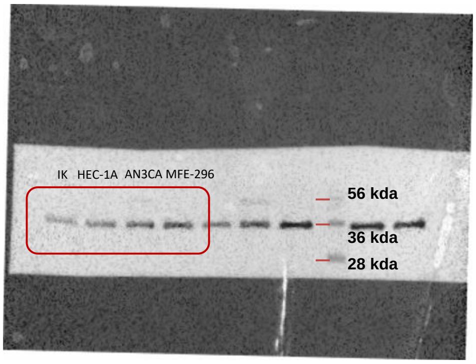

Figure-S3 B (Left)

ADI 1

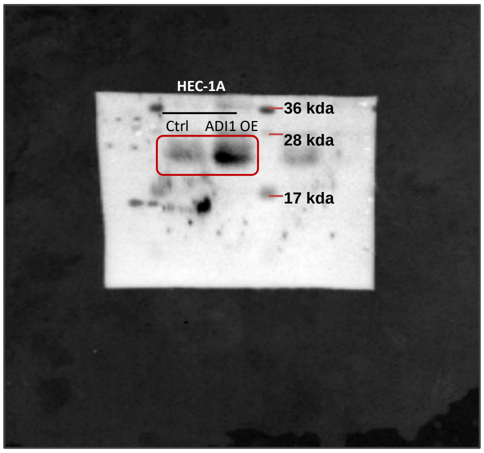

GAPDH

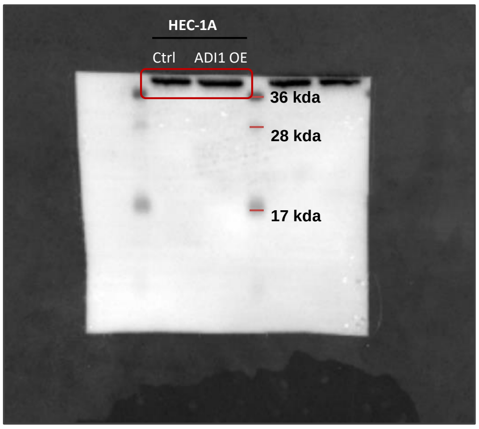

Figure-S3 B (Right)

ADI 1

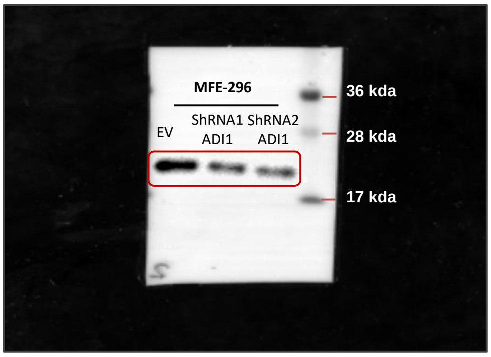

GAPDH

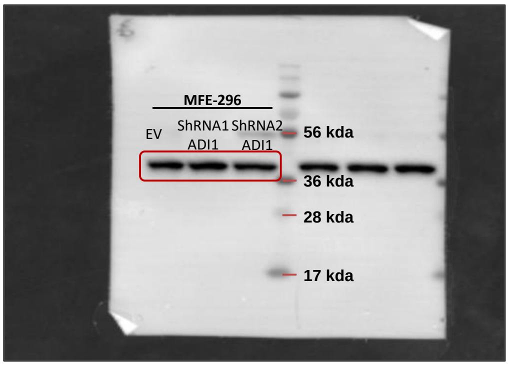

Figure-S4 B

β-catenin

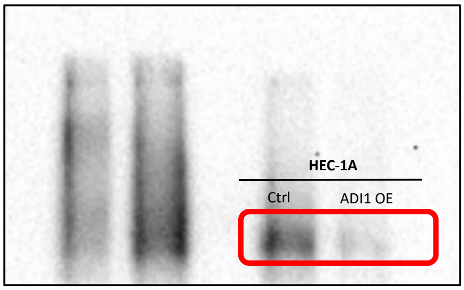

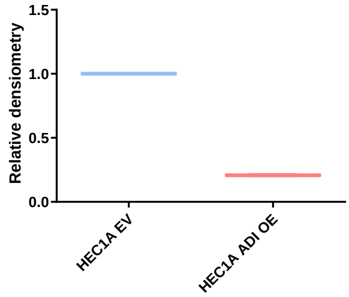

E-cadherin

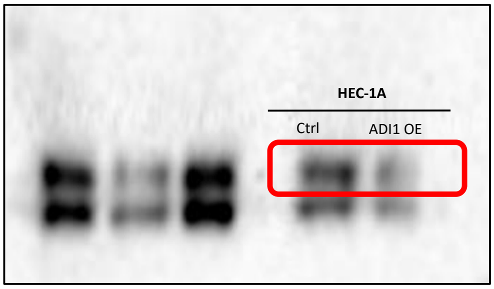

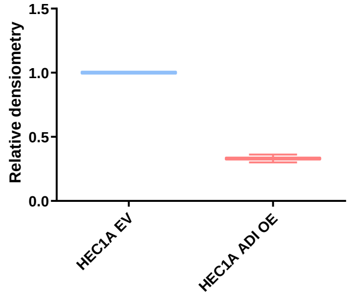

Vimentin

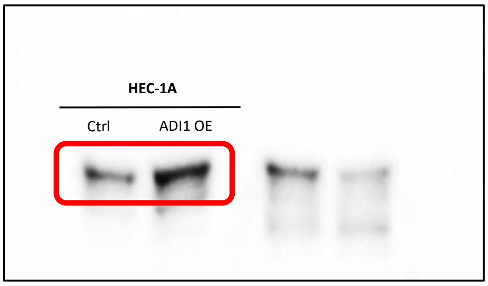

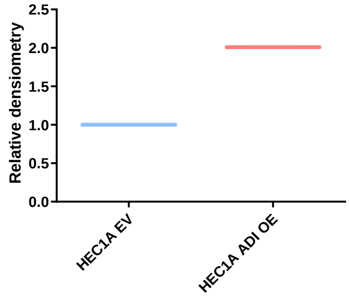

SNAIL

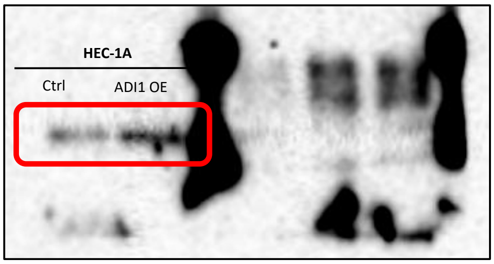

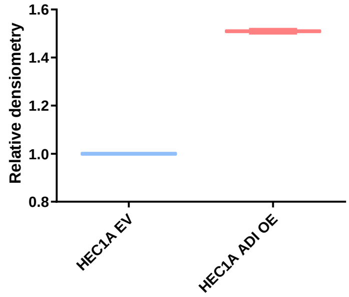

GAPDH

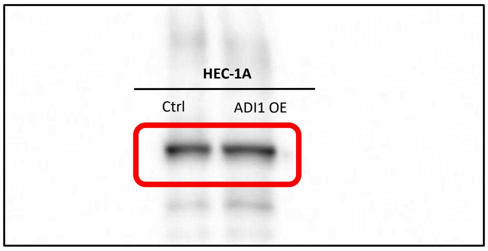

The quantification of the densiometric intensity of each western blot ADI1 band has been calculated from three western blot plots from three independent experiments. GAPDH was used as loading control.

Colorimetric- Uncropped western blots

Figure-S4 B

β-catenin

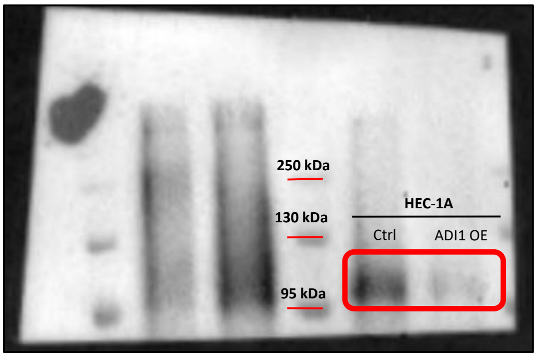

E-cadherin

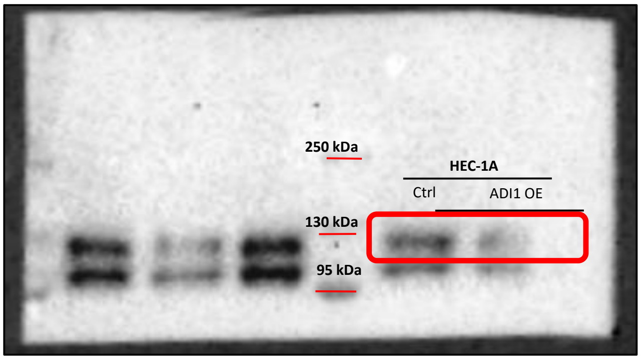

Vimentin

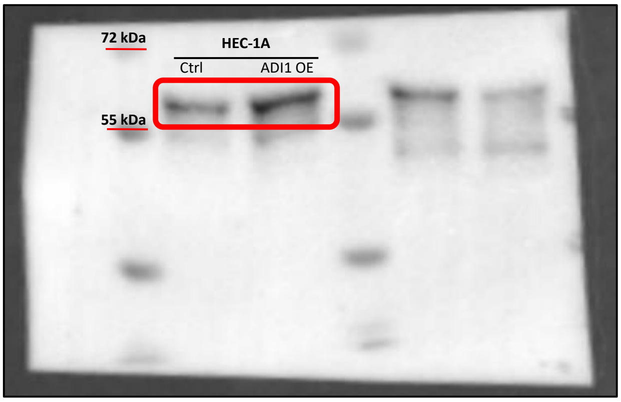

SNAIL

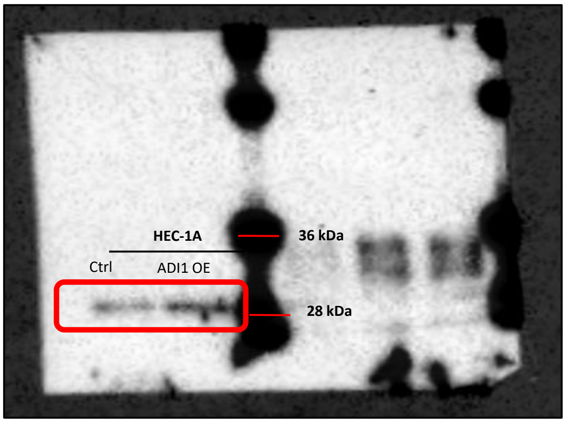

GAPDH

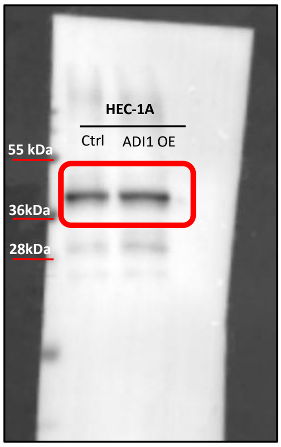

Figure-S4 D

β-catenin

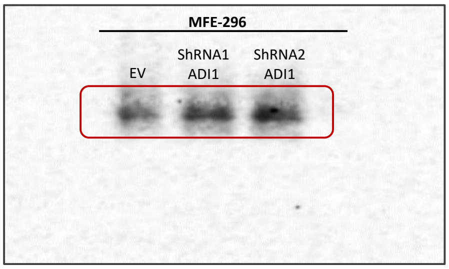

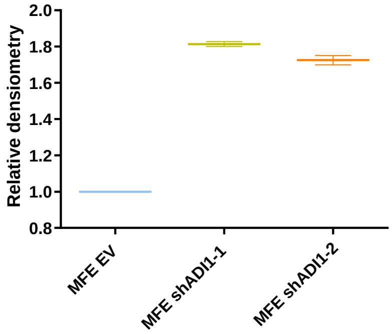

E-cadherin

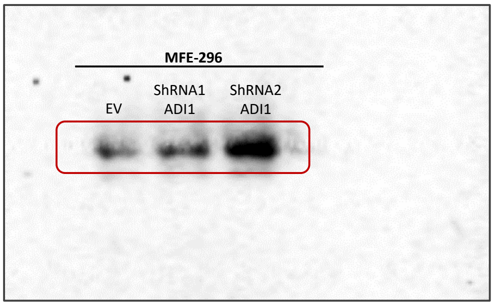

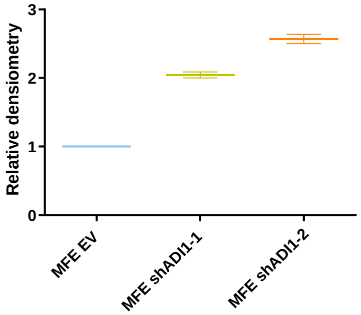

Vimentin

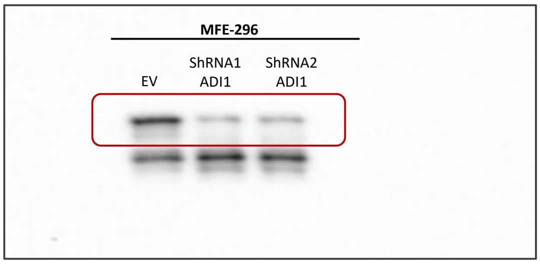

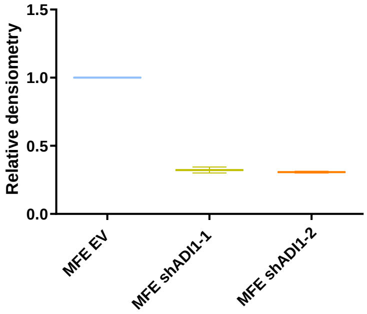

SNAIL

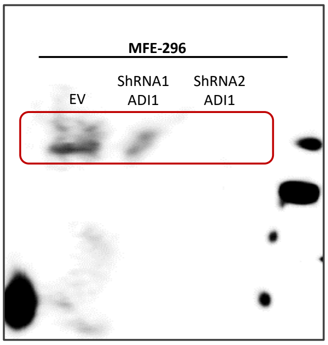

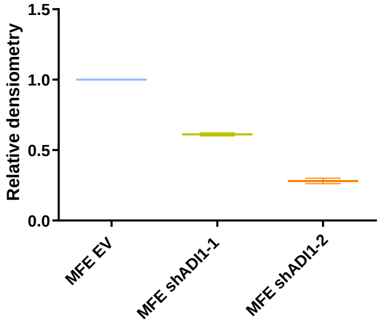

GAPDH

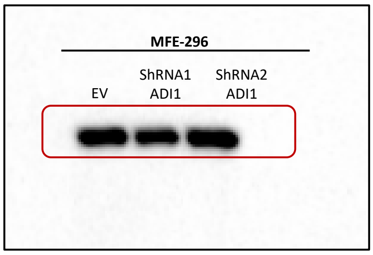

The quantification of the densiometric intensity of each western blot ADI1 band has been calculated from three western blot plots from three independent experiments. GAPDH was used as loading control.

Figure-S4 D

β-catenin

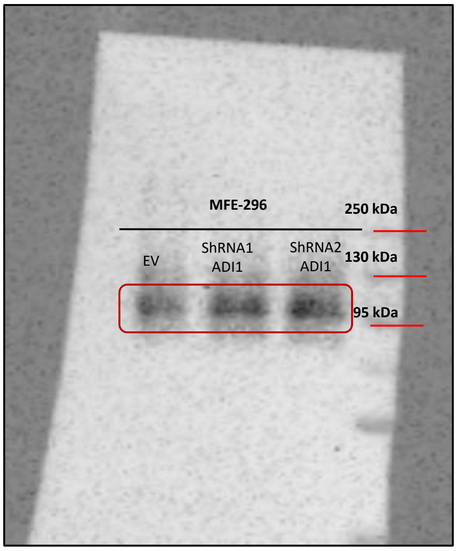

E-cadherin

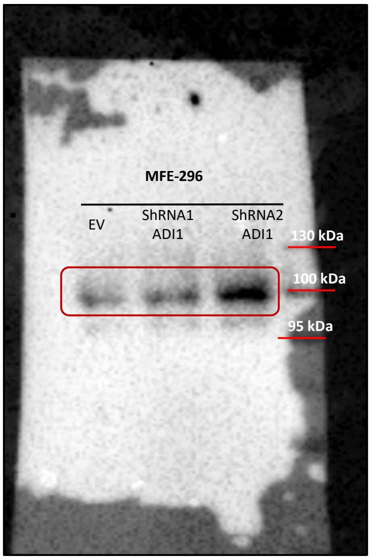

Vimentin

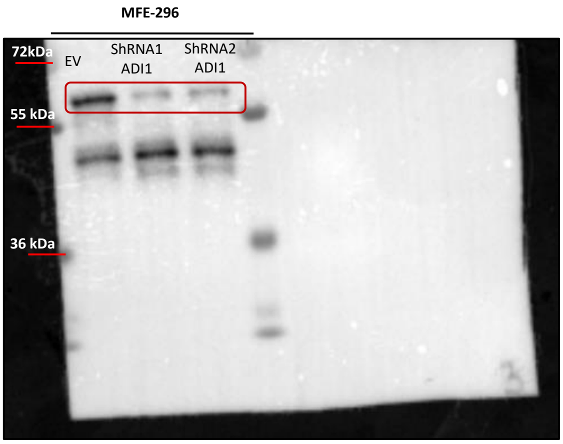

SNAIL

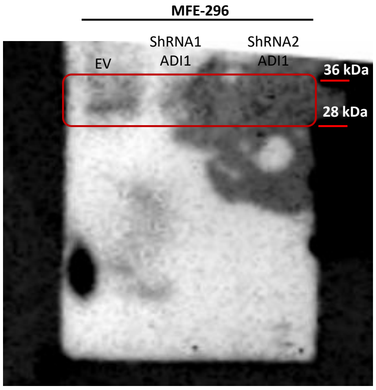

GAPDH

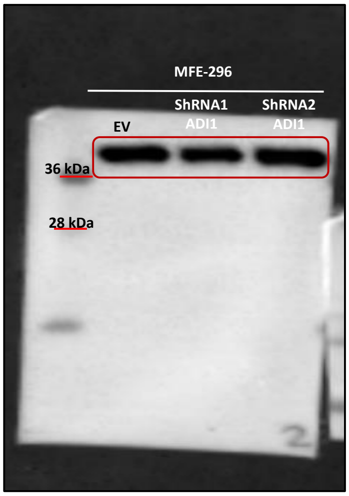

Supplement: Supplementary file 1 [file cancers-14-02842-s001.zip › 1736004-supp/File S2.pdf]
